# Enhanced Antimicrobial Activity of Ciprofloxacin Encapsulated in Sophorolipid-Based Nano-Assemblies Against Ciprofloxacin-/Methicillin-Resistant *Staphylococcus aureus* (MRSA)

**DOI:** 10.3390/pharmaceutics18010104

**Published:** 2026-01-13

**Authors:** Ankita Jain, Navjot Kaur, Shobit Attery, Hemraj Nandanwar, Mani Shankar Bhattacharyya

**Affiliations:** 1 Biochemical Engineering Research and Process Development Centre (BERPDC), CSIR Institute of Microbial Technology (IMTECH), Chandigarh 160036, India; jain.ankita1712@gmail.com (A.J.); navjotkaur@imtech.res.in (N.K.); 2 Academy of Scientific and Innovative Research (AcSIR), Ghaziabad 201002, India; shobit265@gmail.com (S.A.); hemraj@imtech.res.in (H.N.); 3 Clinical Microbiology and Antimicrobial Research Laboratory, CSIR Institute of Microbial Technology, Chandigarh 160036, India

**Keywords:** ciprofloxacin, SL, niosome, biosurfactant, MRSA

## Abstract

**Background:** Drug delivery against ciprofloxacin-resistant microbial strains is one of the most challenging areas of research in the pharmaceutical industry. The broad-spectrum antibiotic ciprofloxacin often faces challenges due to its poor bioavailability; thus, the activity of this drug is generally compromised against resistant strains. Traditional drug delivery systems, such as liposomes, are utilized to address this issue; however, niosomes have surfaced as a promising successor to their liposomal counterparts due to their superior attributes, such as enhanced stability and reduced toxicity. However, owing to environmental and toxicological concerns over commonly used chemical surfactants in niosomes, there is a pressing need to explore greener and safer alternatives. This study is focused on the application of sophorolipids (SLs), a biosurfactant that is synthesized by the yeast *Starmerella bombicola*, as a vesicular assembly for ciprofloxacin encapsulation. **Methods:** The SL-based niosomal formulation was characterized for particle size, zeta potential, and polydispersity index (PDI), while transmission electron microscopy (TEM) was employed to determine the morphology of niosomes. Agar well diffusion, broth dilution, and biofilm inhibition assays were performed to assess efficacy. **Results:** The niosomal formulations were successfully prepared; among them, the (+)vely charged formulation exhibited a more organized morphology, and their size and zeta potential values were found to be around ~371 nm and 63 mV for the blank niosomes (without the loaded drug) and ~269 nm and 51 mV for the ciprofloxacin-loaded niosomes. The minimum inhibitory concentration and biofilm inhibitory concentration against the MRSA strain were 5 µg/mL and 25 µg/mL, respectively, for the ciprofloxacin-loaded, (+)vely charged SL niosomes—for free ciprofloxacin these values were 40 µg/mL and 100 µg/mL—presenting remarkable potential for biofilm inhibition. **Conclusion:** This study highlights the promising therapeutic potential of SL-based ciprofloxacin-loaded niosomes against the emerging health threat of the MRSA strain.

## 1. Introduction

Methicillin-resistant *Staphylococcus aureus* (MRSA) strains have become a significant concern in public health due to their resistance to multiple antibiotics, highlighting the necessity for innovative approaches in treatment. Ciprofloxacin stands as a potent broad-spectrum antibacterial agent renowned for treating a plethora of bacterial infections, such as infections of the respiratory tract, skin issues, bone and joint infections, urinary tract infections, and infections caused by typhoid-causing bacteria, etc. [1]. Ciprofloxacin is also employed for the treatment of MRSA infections; however, MRSA strains are known to accrue resistance to ciprofloxacin rapidly [2]. One major reason for the resistance to ciprofloxacin is the formation of biofilm as a protective layer. Therefore, investigations into the synthesis of novel compounds to overcome antibacterial resistance and to treat microbial biofilm-associated conditions have emerged as a priority research area today [3]. However, working on existing antibiotics using new techniques for their utilization or increasing their efficacy against MRSA is advantageous in many ways.

Numerous drug delivery systems, including liposomes, immunoglobulins, polymeric micelles, serum proteins, microspheres, and nanoparticles, have been developed to optimize drug delivery and reduce adverse effects. Among these, liposomes have gained prominence, especially for enhancing the therapeutic index of antimicrobial agents [4]. For instance, ciprofloxacin has been encapsulated in liposomes to explore its enhanced solid-state properties, drug dissolution, and release profiles [5,6]. However, niosomes have emerged as a potentially superior alternative to liposomes owing to their enhanced stability, minimal aggregation or precipitation, ease of handling, elevated biocompatibility, high permeability, biodegradability, and diminished toxicity [7,8]. Traditional niosome formulations predominantly employ chemical surfactants, such as Tween and Span, raising significant toxicological and environmental concerns [9]. For instance, the surfactant Tween 20 has been shown to induce apoptosis in target cells despite being considered a safe non-ionic surfactant [10]. Similarly, Span, another chemical surfactant used in traditional niosomal formulation, has been reported to cause hemolysis in red blood cells [11]. SL, a glycolipid biosurfactant primarily produced by *Starmerella bombicola*, consisting of β-1,2-bound glucose units and hydroxylated long-chain fatty acids [12], has emerged as a sustainable alternative to its chemical counterpart (surfactant). A comparative toxicological study of different commercially available surfactants, including Tween 20 and an SL with an open chain (acidic SL), reported SL as having 100–250 times less toxicity against the THP-1, Raw 264, and HaCaT cell lines [13]. With their diverse structural range and applications in various fields, from human cervical cancer treatment to bioremediation, SLs (SLs) offer a promising environmentally friendly alternative for many applications. SL micelles encapsulating curcumin exhibited their role in quorum quenching activity against *Pseudomonas aeruginosa* [14]. Similarly, an SL-based micelle was prepared for the observation of micelle docking in model membranes and cells [15]. An SL niosomal formulation containing Amphotericin B is also known to exhibit excellent effects against *Candida albicans* and its biofilm formation [16]. These studies have shown the ability of SLs to form vesicles that can incorporate desirable drug molecules.

In this study, we report the preparation of a microbe-origin SL-based niosome for the entrapment of ciprofloxacin hydrochloride, thus improving the bioavailability of ciprofloxacin. The formulation has been assessed for effectiveness and efficacy against clinical samples of the ciprofloxacin-resistant MRSA strain. The niosomal formulation of ciprofloxacin was also investigated for its role in the inhibition of biofilm formation by MRSA. We also tried to understand the status of the membrane structure of the MRSA strain due to the interaction with the SL-based niosomal formulation of ciprofloxacin. This study provides a novel way of using microbial biosurfactants for drug delivery vehicles rather than chemical surfactants.

## 2. Materials and Methods

### 2.1. Materials

#### 2.1.1. Chemicals

Ciprofloxacin hydrochloride monohydrate was purchased from Himedia, Mumbai, India. Stearylamine, dicetylphosphate, yeast extract powder, malt extract powder, peptone, dextrose/glucose, agar powder, nutrient agar, and cholesterol were also obtained from Himedia, India. Solvents such as the chloroform and methanol used in the study were of the highest purity. Phosphate-buffer saline (PBS) (1X) and saline solution were prepared in the laboratory.

#### 2.1.2. Organisms, Media, and Growth Conditions

For the investigation of ciprofloxacin-loaded niosomes against MRSA, different strains were selected for examination. They were collected from the Government Medical College and Hospital, Chandigarh, India, and ATCC, USA. To initiate the bacterial culture, each strain was first streaked onto Luria–Bertani (LB) agar plates. After adequate growth, a single, isolated colony was carefully picked from the plate. This colony was then inoculated into 10 mL of LB broth and allowed to grow at 37 °C in an orbital shaker incubator (200 rpm). After 16 h incubation, the bacteria were sub-cultured for an additional 3 h to ensure that the cells were in the exponential growth phase.

#### 2.1.3. SL Strain and Culture Conditions

The glycerol stock of the SL-producing strain, *Starmerella bombicola* MTCC 1910, was received from the Microbial Type Culture Collection (MTCC) at CSIR-IMTECH, Chandigarh, India. To revive this strain, the frozen glycerol stock was plated on YPD agar medium, which comprises 1% yeast extract, 2% peptone, 2% glucose, and 2% agar. After the revival of the strain, a loopful of culture was carefully inoculated into YPD broth. The broth culture was then incubated for two days at 30 °C, 200 rpm, ensuring the optimal growth of the *Starmerella bombicola*.

### 2.2. Methods

#### 2.2.1. Production of SL

The production of SL was carried out as described by Haque et al. [17] with little modification. Briefly, 2% of the 48 h grown culture (inoculum) was transferred to a previously optimized SL production medium consisting of glucose (100 g/L), malt extract (10 g/L), peptone (3 g/L), urea (1 g/L), KH_2_PO_4_ (1.5 g/L), Na_2_SO_4_ (1.5 g/L), and cottonseed oil (100 g/L), cultured at 30 °C, 200 rpm, for 7 days.

#### 2.2.2. Extraction of Crude SL

After the production of the SL, the culture was harvested and centrifuged at 6000 rpm for 20 min. The supernatant was taken out and mixed with hexane for the removal of unutilized oily contents. Further, crude SL from the broth was extracted by using ethyl acetate, and concentrated by using a rotary evaporator at 40 °C. 

#### 2.2.3. Purification and Characterization of Crude SL

The crude SL contained different species of SL. The acidic and lactonic SL was purified from the crude SL by using silica gel column chromatography [16]. Briefly, 50 g silica with a mesh size of 60–120 was mixed with hexane and packed into a glass column (50 × 5 cm). Eluent (250 mL, chloroform/methanol) was run through the column before loading the crude SL. A weighed amount (350 mg) of crude SL was mixed with 400 mg of silica in ethanol and evaporated under a reduced pressure at 40 °C using a rotary evaporator. Dried silica with crude SL was loaded into the column. Different fractions (1–10% of chloroform and methanol) were run through the column, and eluents were collected, dried, concentrated, and analyzed by HPLC (Shimadzu, Japan). In a typical HPLC analysis, crude SL was injected into a reverse-phase C-18 HPLC column (5 µm, 4.5 × 250 mm, Merck, Mumbai, India) and eluted at a flow rate of 1 mL/min, with an eluent system comprising a solvent system of acetonitrile and water (20:80) and detected at 207 nm.

#### 2.2.4. Alkaline Hydrolysis of SL

To obtain the acidic SL, alkaline hydrolysis of the lactonic part was performed. Briefly, 20 g of crude SL was mixed with NaOH (5 M, 50 mL) under stirring and reflux at 90 °C for 10–15 min. After that the pH was adjusted to 4 using a concentrated HCl solution. An equal volume of pentanol was mixed with the reaction mixture and kept under continuous stirring for 24 h. After that, the pentanol layer was removed and kept at −20 °C, overnight. Crystalline acidic SL was obtained at the bottom of the flask, and collected by filtering. The obtained acidic SL was analyzed by RP-HPLC and LC-MS to establish its purity and molecular mass, respectively.

#### 2.2.5. Preparation of Blank and Ciprofloxacin-Loaded Niosomes with Different Charges

The thin-film hydration technique was used for the preparation of the niosomes by mixing the key ingredients, i.e., a charge inducer, cholesterol, and SL [18]. Stearylamine (SA) served as the (+)ve charge inducer while dicetyl phosphate (DCP) was chosen as the (-)ve charge inducer. The ingredients were dissolved in a solvent (10 mL) containing chloroform and methanol in a ratio of 4:1. After complete dissolution of the constituents through meticulous pipetting, thin layer formation was achieved by carefully evaporating the solvents through rotary evaporation at 45 °C, with a rotation speed of 70 rpm. Subsequent to the film formation, hydration was initiated by using a 0.9% NaCl solution. Ciprofloxacin was added during this hydration to become incorporated within the niosome [16]. To achieve niosomes of optimal size, the entire suspension was sonicated in a bath sonicator for 1 h. Finally, we obtained a suspension of ciprofloxacin-loaded niosomes which was further subjected to characterization and used for antimicrobial activity testing.

#### 2.2.6. Characterization of Prepared Niosome

##### Vesicle Size, Charge, and PDI

The Dynamic Light Scattering (DLS) method was employed to determine the size of the particles and a zetasizer was used to measure the zeta potential of the formulated niosomes. For evaluation, the formed niosomes were diluted 10 times using deionized water. That ensured the prevention of multiple scattering phenomena during size analysis. In a similar fashion, the zeta potential was also determined using samples of 100 times dilution.

##### Entrapment Efficiency of Ciprofloxacin-Loaded Niosome Formulations

The entrapment efficiency of ciprofloxacin within niosome formulations was carried out to determine the amount of the encapsulated drug. In a typical procedure, the niosome suspension was centrifuged at 14,000 rpm for 1 h. After the centrifugation the supernatant was harvested to quantify the unencapsulated drug, while the pellet was subjected to two successive washes using normal saline (each at 14,000 rpm for 30 min). Quantitative analysis of the supernatant was performed via UV spectrophotometry at λ 278 nm, employing the blank niosomes as the control. The drug concentration was determined using a standard curve prepared in the identical solvent, and the entrapment efficiency was calculated by using the following formula:

Entrapment efficiency (EE%) = (Initial Drug − Unentrapped drug/Initial drug) × 100(1)

##### Transmission Electron Microscopy (TEM)

To determine the morphology of the niosomes, transmission electron microscopy (TEM) was performed. Briefly, one drop of formulated niosome suspension was deposited on a carbonated copper grid and allowed to dry for 10 min. After 20 min, phosphotungstic acid (PTA) was applied to the samples; finally, the sample was air-dried, mounted, and observed with a Gatan Erlanshen side-mounted camera in Jeol (Japan) JEM2100CR TEM at HT 200KV accelerating voltage with the Lab_6_ filament as an electron source.

#### 2.2.7. Antibacterial Activity of the Niosomes

The antibacterial activity of the prepared niosomes was performed by using the agar well diffusion method, the broth microdilution method, and by determining the biofilm inhibitory concentration (BIC).

##### Agar Well Diffusion Method

To assess the antimicrobial activity of different drug formulations against the MRSA-4 strain, the agar well diffusion assay was employed. The MRSA-4 strain was freshly cultured overnight at 37 °C, followed by a secondary culture. Further, ~1.5 × 10^6^ CFU/mL from the culture was combined with LB agar medium right before it solidified and the medium was poured into Petri plates. Using a gel borer, uniformly spaced wells were created in the agar medium. The ciprofloxacin-loaded niosomes, blank niosomes, and free ciprofloxacin were individually added into separate wells and kept at 4 °C for an hour, allowing them to diffuse. The plates were further incubated at 37 °C for 24 h to visualize the zones of inhibition around each well. The diameters of the zone of inhibition around each well were measured to determine the efficacy of each formulation against MRSA.

##### Spot Assay

To evaluate the susceptibility of MRSA-4 to various formulations, culturing was initiated by inoculating a pure colony of MRSA-4 into 5 mL of LB broth. This culture was subsequently incubated at 37 °C for 18–24 h, reaching turbidity consistent with a 0.5–2 OD. Serial dilutions of this culture were then prepared, yielding bacterial concentrations ranging from 10^−1^ to 10^−8^ CFU/mL. In the meantime, working solutions for the ciprofloxacin, blank niosomes, and ciprofloxacin-loaded niosomes were prepared from their respective stock solutions. The agents were diluted to achieve the intended concentrations of 5, 10, and 20 µg/mL.

For the assay, 5 µL of each bacterial dilution was systematically spotted onto the appropriate LB agar plate. Following this, 5 µL of the corresponding drug concentration was spotted next to the bacterial spot, ensuring no overlap between the two. The plates were then left undisturbed to dry for about 10–15 minutes. After drying, the plates were incubated at 37 °C for 24 h.

##### Minimum Inhibitory Concentration (MIC) Determination

Following the determination of the effective ciprofloxacin-loaded niosome formulation, specifically the (+)vely charged ciprofloxacin-loaded niosomes, against the MRSA-4 strain, the minimum inhibitory concentrations (MICs) of these formulations were determined. This assessment extended to not only the MRSA-4 strain but also included several other strains of methicillin-resistant *Staphylococcus aureus* (MRSA) and methicillin-sensitive *Staphylococcus aureus* (MSSA). The MIC values were determined using the serial microdilution method.

To evaluate the antimicrobial efficacy of different drug formulations against selected strains, the MIC of the ciprofloxacin-loaded niosomes, blank niosomes, and free ciprofloxacin was determined using a 96-well plate-based assay. Firstly, the culture of each strain was grown in MHA (Muller Hinton agar) plates at 37 °C overnight. The next day, the bacterial suspension was adjusted to achieve turbidity equal to that of the 0.5 McFarland standards, giving an approximate concentration of 1 × 10^6^ CFU/mL. For the assay, a serial dilution of each drug formulation, including the ciprofloxacin-loaded niosomes, blank niosomes, and free ciprofloxacin, was prepared directly in the wells of the 96-well plate using CA-MHB broth medium. This ensured a gradient of concentration for each test formulation. Following this, an equal volume of the adjusted bacterial suspension was added to each well and incubated at 37 °C for 18–24 h. After the incubation period, the MIC was established by the optical density (OD) of each well at 600 nm using a spectrophotometer. The concentration at which the OD showed a significant reduction compared to the growth control indicated the MIC.

#### 2.2.8. Biofilm Inhibitory Concentration (BIC) Determination

##### Crystal Violet Assay for Biofilm Quantification

To investigate the extent of biofilm formation, the overnight-grown culture of the MRSA-4 strain was diluted to a ratio of 1:100 into fresh LB media containing 1% glucose. In each well of a 96-well plate, 100 μL of the diluted culture and various concentrations of free ciprofloxacin and niosome formulations (ciprofloxacin-loaded and blank) were added and incubated at 37 °C for 24 h.

To assess biofilm formation, the crystal violet (CV) staining method was used, as described by George [19] with minor modifications. In brief, the planktonic cells were first removed, leaving cells embedded in biofilm, which were subsequently immersed in methanol for 15 min and left to air-dry at room temperature. The biofilm was then stained with a 0.1% CV solution for 15 min, followed by careful washing with MQ water to remove excess stain and dried thoroughly.

To quantify the biofilm, it was suspended in 200 µL of 30% acetic acid. From each well of the original plate, 100 µL of this biofilm suspension was transferred to a new plate added with 100 µL of 30% acetic acid and the absorbance was measured at 595 nm. The percentage of biofilm formation was determined using the formula

% Biofilm = (OD595 of treated cells/OD595 of untreated cells) × 100(2)

##### XTT-Based Quantification of Biofilm Inhibition

To determine the biofilm inhibitory concentration (BIC) against the MRSA-4, strain was initially cultivated in LB broth until it reached a logarithmic phase. Following this, the bacterial suspension was adjusted to an optical density corresponding to a 0.5 McFarland standard. A range of concentrations for each formulation, including ciprofloxacin-loaded niosomes, blank niosomes, and free ciprofloxacin, were prepared. In total, 100 µL of the adjusted bacterial suspension was combined with an equal volume of each drug concentration in each well of the 96-well plate and incubated at 37 °C for 24 h for biofilm formation. After the incubation, the planktonic cells were discarded. The wells were washed thrice with phosphate-buffered saline (PBS) to remove any non-adherent cells. The biofilms formed and were fixed by treating with 200 µL of methanol in each well, followed by incubation for 15 min. After fixing, the methanol was discarded, and the plates were left to dry. To initiate the XTT reduction assay, 100 µL of freshly prepared XTT solution was added to each well, and the plates were incubated in the dark for 2 h at 37 °C. After the incubation, 100 µL of solution from each well was transferred to the wells of a fresh 96-well plate. The absorbance of the formazan product was measured using a 96-well plate reader at a wavelength of 595 nm. The concentration at which a notable reduction in absorbance was observed, compared to the control wells, was considered to be the BIC for each drug formulation. The experiment was conducted in triplicate to ensure accuracy and repeatability.

##### Visualized Biofilm Inhibition Assay Under Confocal Microscopy

To study biofilm inhibition via confocal microscopy, coverslips were f coated with poly-l-lysine, as described by Haque et al. [17] and placed in a 6-well plate. For the development of biofilm, mid-log-phase MRSA-4 cells with a 0.01 OD were mixed with 2 mL of 1% glucose-enriched LB broth. To this, different concentrations of ciprofloxacin-loaded niosomes, free ciprofloxacin, and blank niosomes were added. The plate was then incubated at 37 °C for 24 h. After the incubation, the medium was removed, and the biofilms were washed twice with 1X PBS; the biofilms were stained with SYTO 9 (3.34 mM) dye and kept in the dark at room temperature for 30 min. After staining, the excess dye was removed by washing with PBS. The biofilm-adherent cover slips were then carefully mounted on slides for confocal microscopy examination.

##### Congo Red Agar (CRA) Assay

The ability of MRSA-4 strains to form biofilms was assessed using Congo red agar (CRA) culture. The CRA was composed of 50 g/L sucrose, 37 g/L brain heart infusion broth, 20 g/L agar powder, and 8% Congo red indicator. Following the preparation of the culture medium, each of the tested strains was streaked onto the CRA plates and incubated for 24 h at 37 °C. Upon completion of the incubation, the plates were examined for colony coloration. To further understand the biofilm resistance, the MRSA-4 strain was exposed to varying concentrations of free ciprofloxacin, niosome-encapsulated ciprofloxacin, and blank niosomes. To experiment, specific concentrations of these formulations were added to Petri dishes. Once the Congo red agar was poured and solidified, 2 µL of the MRSA-4 culture (with a 0.01 OD) was streaked onto the medium. The plates were subsequently incubated at 37 °C for another 24 h, allowing for clear observation of CRA [20].

#### 2.2.9. Membrane Permeabilization Assay via Propidium Iodide Uptake

The integrity of MRSA-4 cells was determined through PI staining, post treatment with ciprofloxacin-loaded niosomes, free ciprofloxacin, and blank niosomes. The log-phase MRSA-4 cells, exhibiting a 0.2 OD, were treated with different concentrations of ciprofloxacin-loaded niosomes, free ciprofloxacin, and blank niosomes in a 2 mL LB broth. This mixture was then incubated at 37 °C for 3 h in an orbital shaker at 200 rpm. Following incubation, 1 mL from each sample was centrifuged (at 9000 rpm) for 5 min. The resultant supernatant was discarded, and the cell pellets were washed with 1X PBS buffer. Each pellet was then resuspended in 1 mL of 1X PBS. An aliquot of 250 µL was taken for PI staining where 2 µL of a 50 µg/mL PI dye was added to the each of the samples, ensuring the absence of light exposure and incubated at 37 °C for 15 min. Heat-killed MRSA-4 cells were used as a comparative (+)ve control. Before microscopic examination, the stained samples were washed with PBS to eliminate any residual dye. Finally, the stained cells were visualized under a confocal laser scanning microscope.

#### 2.2.10. Statistical Analysis

Statistical analysis was performed using Microsoft Excel and GraphPad 8 software. Experiments were performed in triplicate and are represented as mean ± standard deviation (SD). The significance of the difference among formulations was estimated by performing ANOVA analysis, and the *p*-value was calculated. A *p*-value < 0.005 is known as a significant difference. Bar graphs were prepared for comparison. Confocal images were presented using NIS viewer 4.50 software. The scale bar is indicated in the images.

## 3. Results

### 3.1. Purification and Characterization of SL

SL was produced by using the yeast *S. bombicola* [17]. The crude product (SL) was purified by column chromatography. A set of elution fractions containing different concentrations of MeOH (1–10%) and water was extracted from the silica column and examined by RP-HPLC. The crude extract contained both lactonic and acidic SLs (Supplementary Figure S1A), which were eluted from the column after separation with different retention times (RTs). The lactonic part was eluted at RT 12.4 min and the acid part was eluted at RT 2.6 min (Supplementary Figure S1B,C). Since the crude extract contained a mixture of both lactonic and acidic SL (Supplementary Figure S2A), the lactonic part was subjected to alkaline hydrolysis to obtain a higher yield of acidic SL. The obtained product was examined by LC-MS. The presence of different species of acidic SLs was confirmed by LC-MS analysis (Supplementary Figure S2B).

### 3.2. Preparation and Characterization of SL Niosomes

**Figure 1:**
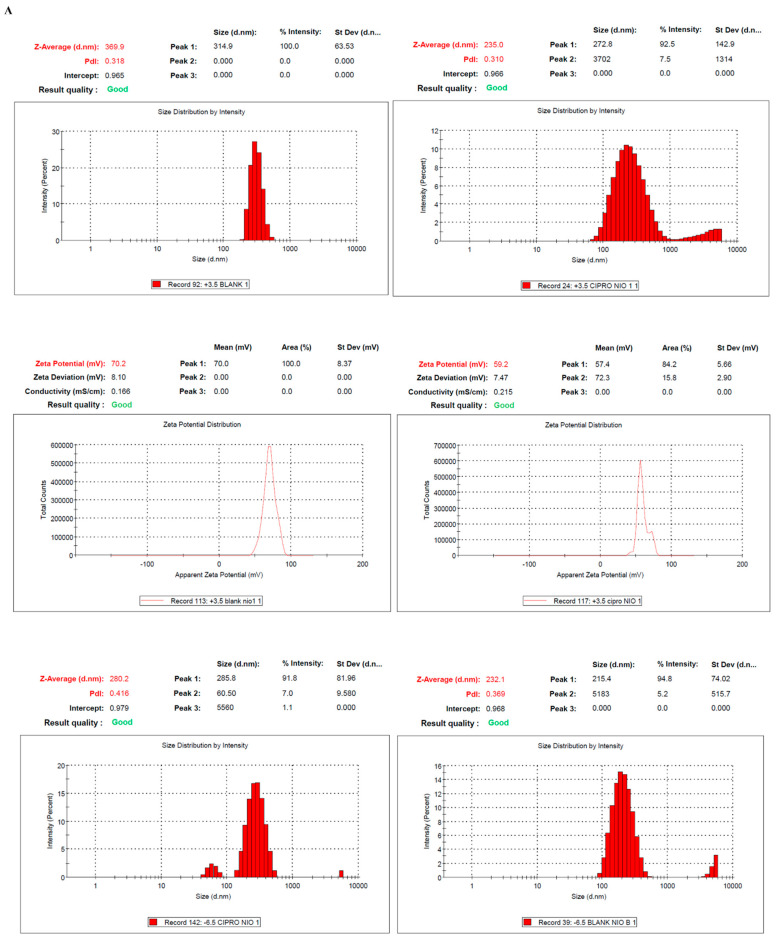

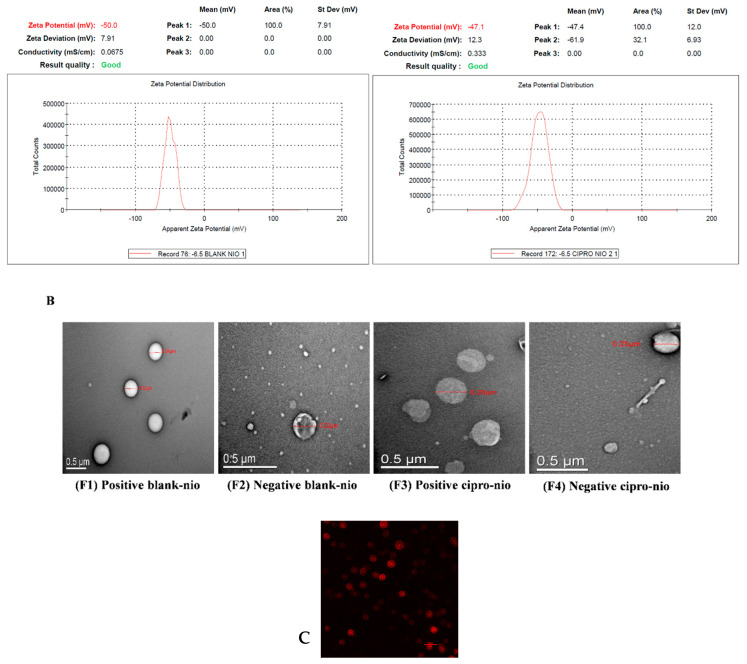
Measurement of size, zeta potential, and morphology of different niosomal formulations: (**A**) DLS (upper panel) and zeta potential (lower panel) of F1: (+)vely charged blank niosomes, F2: (-)vely charged blank niosomes, F3: (+)vely charged ciprofloxacin niosomes, and F4: (-)vely charged ciprofloxacin niosomes (from left to right). (**B**) Transmission electron micrograph (TEM) of different niosomal formulations showing their respective morphologies and (**C**) Nile red-stained (+)vely charged niosomes visualized under confocal laser microscopy (bar scale—10 μm).

For the preparation of SL niosomes of different charges, stearylamine (SA) and dicetyl phosphate (DCP) were used as the (+)ve and (-)ve charge-conferring agents, respectively. A series of niosomes with different combinations of charge-conferring agents was prepared to encapsulate ciprofloxacin. The conditions for the preparation of niosomes were optimized based on size, stability, and polydispersity. A concentration of (3.5 mg) SA and (6.5 mg) DCP was found to be optimum for niosome preparation (Table S1, Supplementary Information). The particles were characterized for their size and zeta potential by using DLS. As shown in Figure 1A, the size of (+)vely charged SL niosomes (without a loaded drug) was found to be around ~371 nm, whereas the size of ciprofloxacin-loaded (+)vely charged SL niosomes was *~*269 nm. In the case of DCP-containing niosomes ((-)vely charged), the size was found to be *~*225 nm, and it was *~*274 nm for blank and drug-loaded niosomes, respectively. The change in zeta potential of the SA-containing niosomes was found to be 63 mV and 51 mV, respectively, for the blank and ciprofloxacin-loaded niosomes. However, the ciprofloxacin-loaded and blank niosomes, that contained DCP, showed zeta potentials of −59 mV and −63 mV, respectively.

#### 3.2.1. Visualization of SL-Based Niosomal Formulation

The niosomes were visualized by using TEM. The formed niosomes were of spherical shape (Figure 1B); however, the (+)vely charged niosomes (blank and ciprofloxacin-loaded, 1BF1 and 1BF3) were found to have a more organized structure compared to the (-)vely charged (blank and ciprofloxacin-loaded, 1BF2 and 1BF4) niosomes. Further, the (+)vely charged niosomes were co-loaded with Nile red and visualized under a confocal laser scanning microscope. As depicted in Figure 1C, several niosomes exhibiting red fluorescence appeared, with their distinctive spherical shape indicating incorporation of Nile red.

#### 3.2.2. Entrapment Efficiency (EE) of Ciprofloxacin-Loaded SL Niosomes

The entrapment efficiency of ciprofloxacin-loaded SL niosomes was determined by subtracting the amount of unincorporated drug present in the preparation solution. The (+)vely charged niosomes were found to have an entrapment efficiency ≈71%.

### 3.3. Antibacterial Activity

#### 3.3.1. Zone of Inhibition

**Figure 2:**
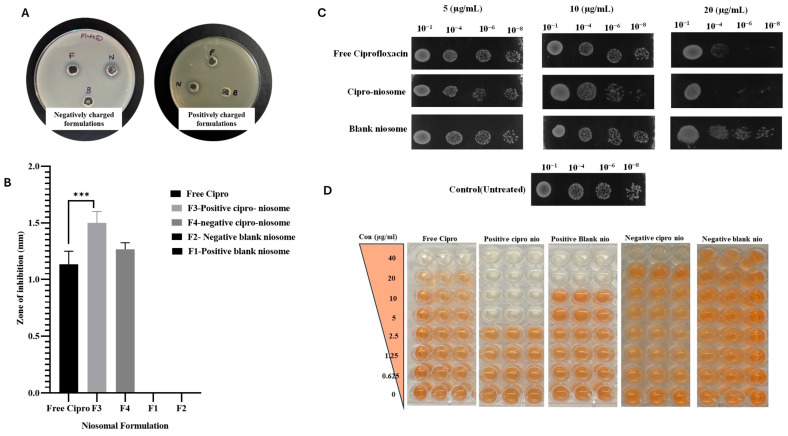
Antibacterial activity of various niosomal formulations against MRSA-4 cells. (**A**) Representative images of the zones of inhibition observed in the agar well diffusion assay. (**B**) Quantitative measurement of the diameter of the zones of inhibition of the niosome formulations. *** represents the level of statistical significance, with a *p*-value < 0.0004. (**C**) Spot assay at 3 different drug concentrations (5, 10, and 20 µg/mL) to assess dose-dependent antibacterial response, and (**D**) determination of minimum inhibitory concentration (MIC) values of free and ciprofloxacin-loaded niosomal formulations.

The susceptibility of MRSA-4 to various treatments was examined using the zone of inhibition assay. Notably, the ciprofloxacin-loaded (+)vely charged niosomes showed significant antibacterial activity by forming a prominent zone of inhibition measuring 15 mm in diameter (Figure 2A,B). Both the ciprofloxacin-loaded (-)vely charged niosomes and ciprofloxacin showed similar zones of inhibition (approximately 11.3 mm). However, the blank niosomes exhibited no antibacterial activity, evidenced by the absence of a zone of inhibition.

#### 3.3.2. Spot Assay of Niosomal Formulations Against MRSA-4

The efficacy of ciprofloxacin-loaded niosomal formulations against MRSA-4 was examined using a spot assay. The obtained result indicated notable enhancement of the antimicrobial activity of the ciprofloxacin-loaded niosomes against MRSA-4 compared to its drug-free counterpart. Complete growth inhibition was observed at a 10 µg/mL concentration of ciprofloxacin-loaded niosomes as compared to free ciprofloxacin with the spot containing 10^−8^ dilutions (Figure 2C). Moreover, pronounced cell growth inhibition was observed surrounding the spots (10^−4^ to 10^−8^ dilution) containing ciprofloxacin-loaded niosomes compared to free ciprofloxacin. The untreated spots and the spots containing blank niosomes did not exhibit any significant antimicrobial activity against MRSA-4.

#### 3.3.3. Minimum Inhibitory Concentration (MIC)

**Figure 3:**
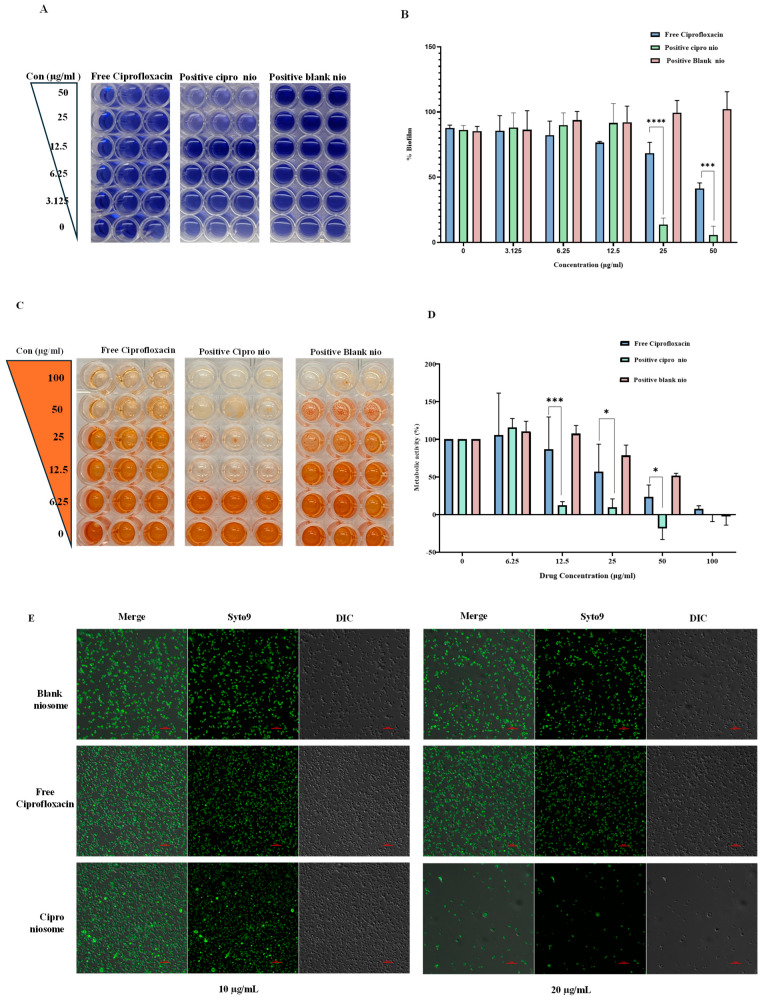

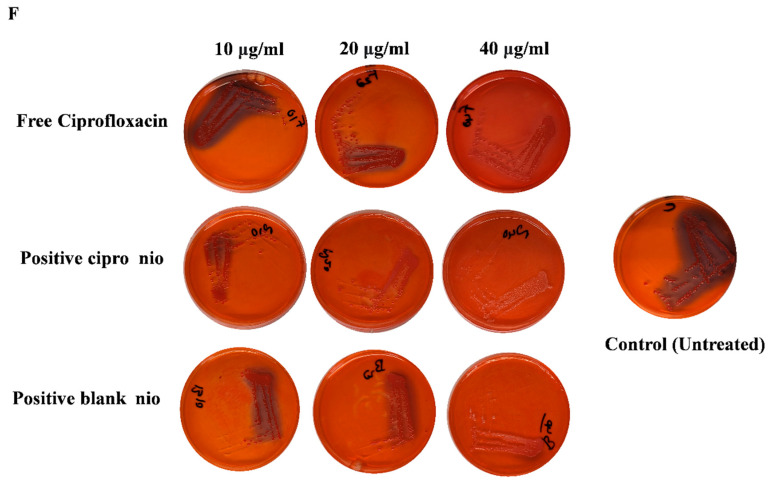
Effect of free and encapsulated ciprofloxacin niosomal formulations on the biofilm of MRSA-4. (**A**) Crystal violet staining of formed biofilm. (**B**) Quantitative analysis of the biofilm showing concentration-dependent inhibition using crystal violet staining (*p* < 0.0001). (**C**) Assessment of metabolically active cells within the biofilm using the XTT assay. (**D**) Quantitative analysis of inhibition of the biofilm formation in a concentration-dependent manner (*p*-value < 0.0003), (**E**) CLSM image showing the effect of free ciprofloxacin and its niosomal formulation at different concentrations (scale bar—10 µm), and (**F**) Congo red agar (CRA) assay highlighting the difference in exopolysaccharide production using ciprofloxacin-loaded niosomes and their drug-free counterpart. All the experiments were performed in triplicate, and the graph was plotted using GraphPad Prism 8 using average values with SD. (*,***, **** represent the level of significance between the free ciprofloxacin and (+)vely charged ciprofloxacin niosomes, calculated using GraphPad Prism 8 (where * *p* < 0.05 represents a significant result, *** *p* < 0.001 represents a very highly significant result, and **** *p* < 0.0001 represents an extremely significant result).

Upon establishing the activity of the ciprofloxacin-loaded (+)vely charged niosomes, the formulation was further checked against different strains of MRSA, collected from different resources. It is evident from Supplementary Table S2 that the (+)vely charged ciprofloxacin-loaded niosomes (Figure 3) showed higher antibacterial efficacy, especially against ciprofloxacin-resistant MRSA strains compared to their free-drug counterpart. The susceptibility of the strains used in this study against different antibiotics is provided in Supplementary Table S3. However, no substantial difference was observed against MSSA, as well as ciprofloxacin-sensitive MRSA strains, when compared with free ciprofloxacin and ciprofloxacin-loaded niosomes. Among the various strains examined, it was observed that the MRSA-4 strain exhibited a significant level of resistance to ciprofloxacin, with a notable MIC of 40 µg/mL.

**Figure 4:**
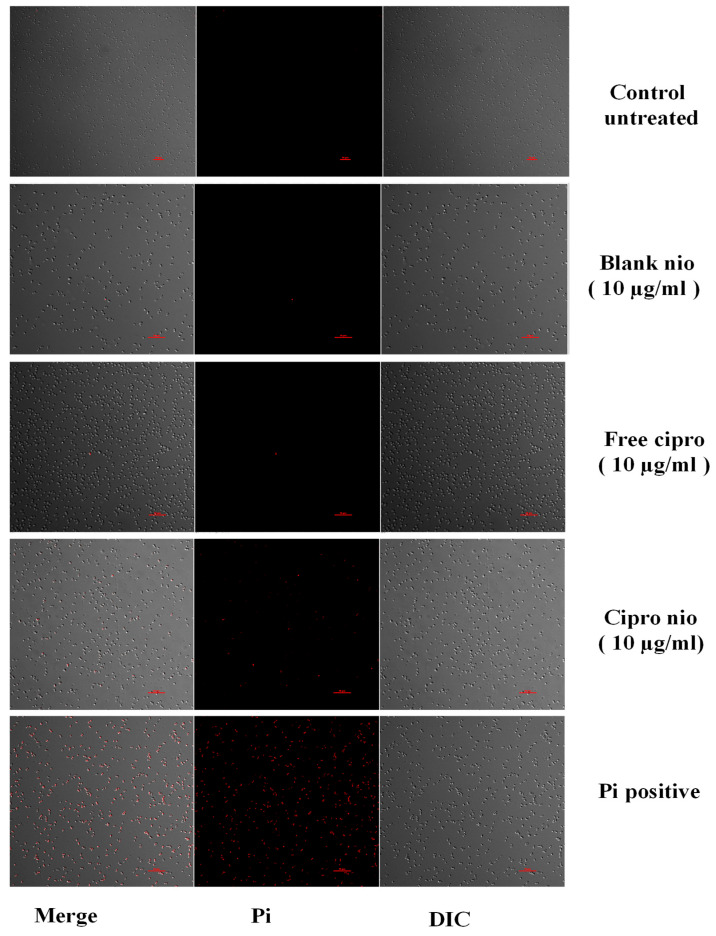
Confocal microscopic images showing compromised membrane integrity in the MRSA-4 strain under treatment with (+)ve ciprofloxacin-loaded niosomes (scale bar—10 µm).

As evident from Figure 2D, ciprofloxacin-loaded (+)vely charged niosomes exhibited superior antimicrobial efficacy against MRSA-4 with an MIC value of 5 µg/mL (Figure 3). In contrast, free ciprofloxacin exhibited an MIC of 40 µg/mL against the same strain. Against other strains, i.e., GMCH 6152, GMCH 3939, and MRSA-1, the MIC values were 20 µg/mL, 10 µg/mL, and 10 µg/mL, respectively. It is worthy of mention that the (+)vely charged blank niosomes (Figure 2) exhibited an antimicrobial effect against MRSA-4 with an MIC value determined to be 20 µg/mL, suggesting that the combination of the niosome-formulating molecules also have some amount of inherent microbial resistance-modulating properties. On the other hand, the (-)vely charged ciprofloxacin-loaded niosomes (Figure 4) showed a similar MIC value (40 µg/mL) as obtained with free ciprofloxacin.

### 3.4. Evaluation of Biofilm Inhibitory Concentration (BIC)

Biofilms provide a defence against antimicrobial compounds. The MRSA strains with the capability of biofilm formation confer a higher resistance to the microorganism. Therefore, the ciprofloxacin-loaded niosomes were tested against the biofilm formation capability of the MRSA-4 strain. The ciprofloxacin-loaded (+)vely charged niosomes showed significant anti-biofilm activity with a BIC of 25 µg/mL. In contrast, the free ciprofloxacin exhibited a BIC of 100 µg/mL. Notably, the (+)vely charged blank niosomes themselves exhibited a biofilm inhibition capacity with a BIC of 100 µg/mL (Figure 3A,B). Figure 3C,D represent the presence of metabolically active cells within the biofilm, assessed through XTT assay. At a 12.5 µg/mL concentration of (+)vely charged ciprofloxacin-loaded niosomes, a significant reduction in the metabolically active cells was observed (<20%). Further, at a 25 µg/mL concentration of the same niosomes, <10% metabolically active cells were observed. In contrast free ciprofloxacin was shown to have a higher BIC (100 µg/mL).

#### 3.4.1. Visualization of Biofilm Inhibition Using Confocal Laser Microscopy

The ability of various ciprofloxacin formulations to inhibit biofilm formation by MRSA-4 was investigated using confocal microscopy. SYTO 9, which labels live bacteria by generating green fluorescence, was used to visualize the bacterial cells within the biofilm. Upon treatment with free ciprofloxacin and (+)vely charged ciprofloxacin-loaded niosomes (20 µg/mL), a noticeable reduction in the fluorescence was observed in the cells treated with (+)vely charged ciprofloxacin-loaded niosomes (Figure 3E), while significant green fluorescence was observed in the cells treated with free ciprofloxacin, indicating the presence of a higher number of live cells remaining within the free ciprofloxacin-treated samples. The observation suggests that the (+)vely charged ciprofloxacin niosomes not only effectively hinder the growth of MRSA-4 cells in the biofilm but also cause substantial cell death.

#### 3.4.2. Congo Red Agar (CRA) Assay

The Congo red agar (CRA) assay was used to assess the ability of ciprofloxacin-loaded niosomes to inhibit biofilm formation by MRSA-4. Notably, black colonies with a dry, crystalline texture signify the production of exopolysaccharide slime (EPS), typical of biofilm architectures. When MRSA-4 cells were exposed to 20 µg/mL free ciprofloxacin, a noticeable reduction in the appearance of black colonies was observed, suggesting reduced biofilm formation (Figure 3F). At a 40 µg/mL concentration of ciprofloxacin, there was a substantial reduction in black-coloured colonies; most of the colonies were red in colour, indicating minimal biofilm production. Whereas the (+)vely charged cipro-niosome-treated (20 µg/mL) colonies were mostly red in colour, only few appeared to be black colour, indicating a lower amount of biofilm formation. The effect of the (+)vely charged ciprofloxacin-loaded niosomes (at 40 µg/mL) appeared to be more prominent. In contrast, the untreated cells did not exhibit any discernible impact on biofilm production, and colonies appeared black [20].

### 3.5. Visualization of Membrane Integrity Under Confocal Microscopy

The membrane permeabilization studies were carried out by using the propidium iodide (PI) uptake assay. When cell membrane integrity is compromised, PI enters the cells and binds with DNA, leading to observable fluorescence. An increase in fluorescence denotes disrupted cell membranes. When cells were visualized under a confocal laser scanning microscope, the untreated control samples of MRSA-4 showed no PI fluorescence, signifying that the cell membranes remained largely intact in the absence of external treatments (Figure 4). However, when the cells were exposed to free ciprofloxacin at a concentration of 10 µg/mL, they exhibited an increase in PI fluorescence, indicating an observable amount of membrane disruption. However, treatment with (+)vely charged ciprofloxacin-loaded niosomes showed the appearance of a higher amount of fluorescence compared to the equivalent concentration of free ciprofloxacin. The cells treated with the blank niosomes (10 µg/mL) presented no PI fluorescence during the microscopic observation.

## 4. Discussion

Ciprofloxacin is one of the most commonly used drugs for the treatment of MRSA infections; however, MRSA strains are known to rapidly acquire resistance to ciprofloxacin [2]. Despite studies being conducted to address the growing challenges of resistance to ciprofloxacin using nano-assemblies, including liposomes, chitosan nano-particles, micelles, and solid lipid nano-particles, this field of research remains an active field of investigation. Recent strategies involve alteration of the types of surfactants used in the assemblies or by changing the ratio of ingredients used in the formation of assemblies, mainly to improve critical parameters such as biocompatibility, environmental friendliness, toxicity reduction, drug loading efficiency, and, most importantly, therapeutic efficacy. In this study, we used thin-film hydration technology [16] for the incorporation of ciprofloxacin into nano-vesicles containing charge-conferring agent(s). A comparative overview of the literature related to ciprofloxacin delivery systems is presented in Supplementary Table S4, highlighting important parameters such as the method of preparation, size, zeta potential, etc. Comparative analysis demonstrated that, while each system offers unique advantages, limitations persist in each system. In the present study, SL was used in niosome preparation, not only leveraging the biocompatible and amphiphilic nature of the surfactant but also helping to achieve enhanced therapeutic potential in terms of biofilm inhibition.

In the present study introduction of surface-charge-conferring agents enhanced the stability of the vesicles and played a crucial role in charge separation and stability [21]. Notably, in all the niosomal formulations, the charges are well distributed (both (-)ve and (+)ve) ensuring the stability of the formulation. In fact, ciprofloxacin contains two ionizable groups, a carboxylic acid (pK_a_ = 6.09) and a secondary amine (pK_a_ = 8.74). The size reduction of the ciprofloxacin-loaded (+)vely charged niosomes may be due to the compaction of the molecules owing to the (+)ve interaction with the loaded drug. The interaction also leads to the reduction in the polydispersity of the particles. Further, the TEM studies also confirmed the morphology, compact organization, and spherical structure of the (+)vely charged niosomes.

The pH of the suspension solution played a crucial role in the efficiency of encapsulation of ciprofloxacin (EE) within the niosomes. The ionized (-)ve charge on the carboxylic group of ciprofloxacin may have been involved in an electrostatic interaction with the (+)vely charged surface agents (SAs) of the niosomes. This may have contributed to the elevated EE% of the drug within the (+)vely charged ciprofloxacin-loaded niosomes. Conversely, the presence of (-)vely charged groups on the niosomal components (DCP) induces electrostatic repulsion, leading to a reduction in the EE% of ciprofloxacin [22]. Similar trends were observed in the integration of charge-inducing agents into the liposoms encapsulating ciprofloxacin [22] and other drugs, such as acetazolamide, azathioprine, and indomethacin encapsulated in liposomes [23,24].

Ciprofloxacin, like other fluoroquinolones, displays unique pH-dependent solubility profile, resembling a ‘U-shaped’ curve. Since the molecule exhibits zwitterionic properties, its solubility increases at both higher and lower pH values compared to its isoelectric point (pH 7.2). When the solution pH is > 7, ciprofloxacin becomes (-)vely charged due to the deprotonation of the carboxylic group; while, at pH < 7, the ciprofloxacin molecules acquire a (+)ve charge through the protonation of the amine group, and around a neutral pH, ciprofloxacin maintains a balanced net neutral charge [25,26]. This unique zwitterionic property of the molecule is characterized by the dual pKa values (approximately 6 and 8.8) of the molecule.

A marked difference in the zones of inhibition between the (+)vely charged ciprofloxacin-loaded niosomes and free ciprofloxacin proved the enhanced efficacy of the formulation. The obtained result also showed the potential therapeutic efficacy of the (+)vely charged ciprofloxacin-loaded niosomes against MRSA strains.

The MIC values were evaluated for different niosomal formulations along with free ciprofloxacin against MRSA and MSSA strains. Particular emphasis was given to conducting further studies on the MRSA-4 strain to gain a comprehensive understanding of the efficacy of the formulation. Drug encapsulation within nano-carrier systems, like niosomes, can potentiate the activity of the molecule by improving cellular uptake and bypassing efflux mechanisms, which are common to the drug resistance strategies employed by MRSA strains [27]. This may explain the reason behind the significant difference in MICs between the ciprofloxacin-loaded niosomes and the free drug. Furthermore, the lipid compositions, including that of the acidic SL, could interact with bacterial membranes, potentially accounting for the observed MIC value [28,29]. The crystal violet staining of the biofilm of the MRSA-4 strain reveals that the treatment with (+)vely charged ciprofloxacin-loaded niosomes caused significant inhibition of biofilm formation. These results indicate that the ciprofloxacin-loaded niosomes could be a promising therapeutic strategy for combating MRSA-4 biofilm-related infections.

Amplification of the antibiofilm potential of the antimicrobial agents encapsulated within vesicular systems may have arisen from the enhanced penetration of the drug into the biofilms leading to an increased disruption of the biofilm matrix [30]. The difference in BICs between the ciprofloxacin-loaded niosomes and their free counterpart indicates this phenomenon. Furthermore, the anti-biofilm effect of blank niosomes might be attributed to the lipid composition, which may have interfered with biofilm structure and stability [31,32].

The CRA assay (Figure 3F) is a qualitative test used for visual assessment of biofilm production by microorganisms. The exopolysaccharides produced by the biofilm-dwelling bacterial cells bind to Congo red, resulting in the appearance of black-coloured colonies, while non-biofilm-producing strains do not bind to the dye, producing colonies that are not black in colour. Monitoring membrane integrity is crucial to validate the effectiveness of ciprofloxacin-encapsulated niosomes in inflicting damage to the bacterial cell membranes. PI staining of the treated cells provided evidence of membrane disruption by the (+)vely charged ciprofloxacin-loaded niosomes.

Studies suggest that ciprofloxacin can induce structural and functional changes in the cell membrane indirectly, by affecting membrane integrity through various mechanisms, including the induction of oxidative stress, the disruption of membrane-associated proteins, and alterations in membrane fluidity [26,33,34,35]. The combined effects of ciprofloxacin on bacterial enzymes, membrane proteins, and oxidative stress contribute to the loss of membrane integrity, leading to bacterial cell death. Thus, a compromised outer structure leads to membrane permeability, allowing the entry of PI into the cells. Furthermore, the use of ciprofloxacin-encapsulated niosomes might have additional effects on the bacterial membrane. The cell walls of gram-(+)ve bacteria are characterized by the presence of a significant amount of anionic polysaccharides (constituting up to 60% of the cell wall composition; these polysaccharides are linked to either the cytoplasmic membrane or the peptidoglycan layer), contributing to the overall (-)ve charge of the membrane [36]. Therefore, the (+)vely charged ciprofloxacin-encapsulated niosomes can effectively interact with these (-)vely charged membranes [37]. This interaction enhances the activity of ciprofloxacin, leading to the improved efficacy of the drug and causing significant loss of membrane integrity compared to free ciprofloxacin.

Ciprofloxacin primarily exhibits its antimicrobial activity by inhibiting bacterial DNA gyrase and topoisomerase IV, crucial to replication, transcription, and DNA repair activity [35]. By disrupting these critical enzymes, ciprofloxacin interferes with bacterial DNA synthesis and causes the suppression of growth and replication of bacteria. In essence, this study underlines the potential of the ciprofloxacin-loaded niosome formulation to disrupt MRSA-4 cell membranes.

## 5. Conclusions

The innovative approach of using biosurfactant SL for niosome preparation and employing the niosomes for ciprofloxacin delivery was thoroughly examined against the MRSA-4. Various research studies have been conducted till now for the enhanced delivery of ciprofloxacin against methicillin-resistant *Staphylococcus aureus*, but chemical surfactants such as Span and Tween were used for the formation of niosome assemblies. In this study, for the first study we employed a biosurfactant SL for the preparation of niosomes encapsulating ciprofloxacin and demonstrated that the MRSA-4 strain is highly susceptible to them compared to free ciprofloxacin. Among the tested niosomal formulations, the (+)vely charged formulation encapsulating ciprofloxacin exhibited a larger zone of inhibition, decreased MIC, and better MRSA activity. Also, these (+)vely charged ciprofloxacin-loaded niosomes were more capable of inhibiting biofilm compared to free ciprofloxacin. Thus, these findings highlight the efficiency of biosurfactant-formed, SA-stabilized niosomes in overcoming the challenges of bacterial resistance.

## Data Availability

Data that support the findings of this study are available from the corresponding author upon reasonable request.

## Supplementary Materials

The following supporting information can be downloaded at: https://www.mdpi.com/article/10.3390/pharmaceutics18010104/s1, The supporting information containing an HPLC chromatogram of SLs; mass spectrum of crude and acidic SLs; and tables containing information on the characterization of various combinations of SL niosomes; MIC of free ciprofloxacin; ciprofloxacin-containing (+)vely charged niosomes and only (+)vely charged niosomes against MRSA strains; and information on cultures used in the study has been provided in the Supplementary Materials. Figure S1: HPLC chromatogram showing SL purified from column chromatography. (A) The chromatogram of the 5% fraction. (B) A purified peak of lactonic SL at the retention time of 12.4. (C) An acidic SL at the retention time of 2.6; Figure S2. (A) Mass spectrum of crude SL and (B) Mass spectra of acidic SL; Table S1. Various combinations of SL niosomes and their characterization; Table S2. Evaluation of MICs of different drugs/formulations against MRSA strains; Table S3. Showing information regarding cultures. Table S4. Tabular comparison of various ciprofloxacin-loaded drug delivery platforms along with SL niosome-based delivery system: physicochemical characteristics and target pathogens. References in Supplementary Materials can be found in [38,39,40,41,42].
