# Supplementary material for: Enhanced Antimicrobial Activity of Ciprofloxacin Encapsulated in Sophorolipid-Based Nano-Assemblies Against Ciprofloxacin-/Methicillin-Resistant Staphylococcus aureus (MRSA)
Source: Pharmaceutics. 2026 Jan 13;18(1):104. doi: 10.3390/pharmaceutics18010104 (PMC12845106; doi:10.3390/pharmaceutics18010104)
Supplement: Supplementary file 1 [file pharmaceutics-18-00104-s001.zip › pharmaceutics-3692928-supplementary.pdf]

Enhanced Antimicrobial Activity of Ciprofloxacin Encapsulated in Sophorolipid-Based Nano-Assemblies Against Ciprofloxacin-/Methicillin-Resistant *Staphylococcus aureus* (MRSA)

Ankita Jain [1], Navjot Kaur [1,2], Shobit Attery [2,3], Hemraj Nandanwar [2,3] and Mani Shankar Bhattacharyya [1,2,*]

[1] Biochemical Engineering Research and Process Development Centre (BERPDC), CSIR Institute of Microbial Technology (IMTECH), Chandigarh 160036, India; jain.ankita1712@gmail.com (A.J.); navjotkaur@imtech.res.in (N.K.)

[2] Academy of Scientific and Innovative Research (AcSIR), Ghaziabad 201002, India; shobit265@gmail.com (S.A.); hemraj@imtech.res.in (H.N.)

[3] Clinical Microbiology and Antimicrobial Research Laboratory, CSIR Institute of Microbial Technology, Chandigarh 160036, India

* Correspondence: manisb@imtech.res.in; Tel.: +91-172-6665313; Fax: +91-172-2695215

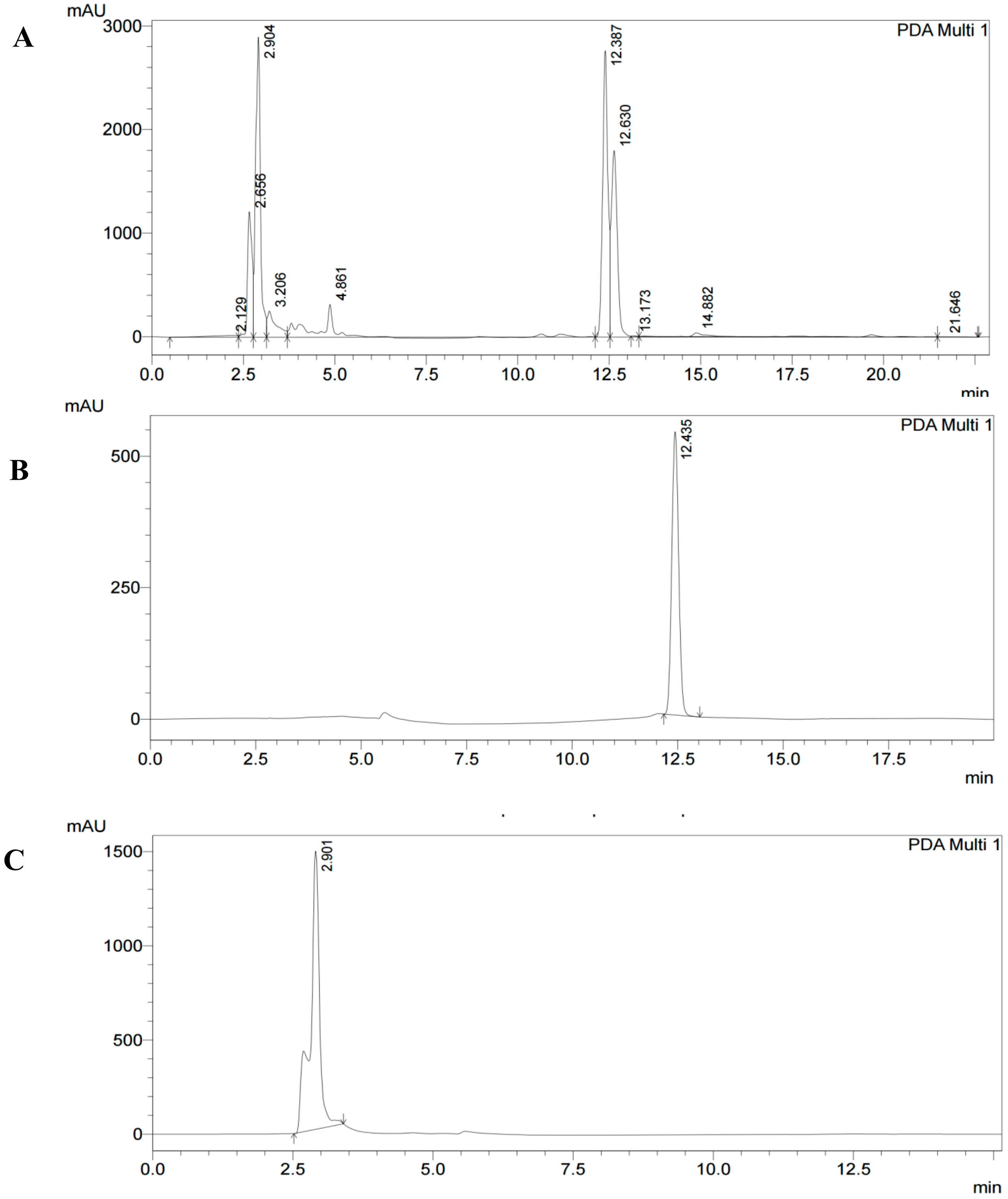

Supplementary Figure S1. HPLC chromatogram showing sophorolipid purified from column chromatography **(A)** The chromatogram of 5% fraction **(B)** A purified peak of lactonic SL at retention time of 12.4. **(C)** An acidic SL at retention time of 2.6

A

B

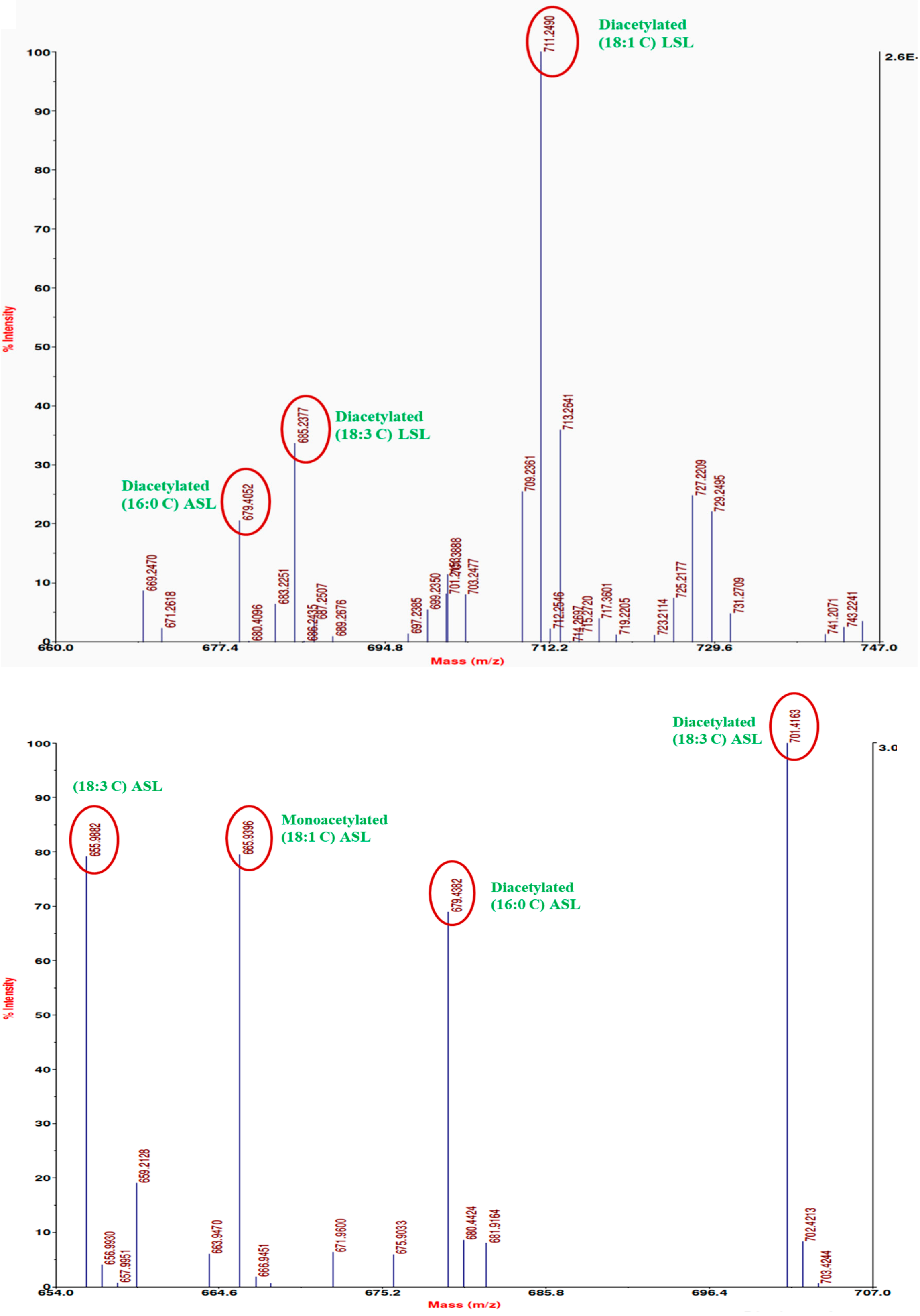

Supplementary Figure S2. (**A**). Mass spectrum of crude sophorolipid. (**B**). Mass spectrum of acidic Sophorolipid

Table

Table S1: Various combinations of SL-niosomes and their characterization

Formulations	Charges (mg)	Cholesterol (mg)	Acidic SL (mg)	Ciprofloxacin (mg)	Size (nm)	Zeta potential (mV)	Poly Dispersity Index (PDI)
F1	STR 3.5	3.5	50	0	371.03 ±27.21	63 ±9.67	0.24 ± 0.11
F2	DCP 6.5	3.5	50	0	225.46 ± 7.14	-63.36±11.60	0.26 ± 0.12
F3	STR 3.5	3.5	50	4	269.9 ± 31.5	51.13 ± 6.99	0.27 ± 0.04
F4	DCP 6.5	3.5	50	4	274.76 ±4.89	-59.86 ± 11.34	0.43 ± 0.03

F1- (+)ve blank niosome, F2- (-)ve blank niosome, F3- (+)ve cipro-niosome, F4- (-)ve cipro-niosome

STR- Stearylamine, DCP- Dicetyl Phosphate

Table S2: Evaluation of MIC of different drugs/ formulations against MRSA strains

S.No.	Cultures name	Free Ciprofloxacin MIC (µg/ml)	Cipro-niosome MIC (µg/ml)	Blank niosomes MIC (µg/ml)
**1.**	ATCC 29213 (MSSA)	0.3	0.3	10-20
**2.**	ATCC 25923 (MSSA)	0.3	0.3	10-20
**3.**	ATCC 43300 (MRSA)	0.3	0.3	10-20
**4.**	ATCC 33591 (MRSA)	0.3	0.3	10-20
**5.**	ATCC-BAA-39 (MRSA)	10	2.5	10-20
**6.**	MRSA-4	40	5	10-20
**7.**	MRSA-1	10	2.5	10-20
**8.**	GMCH 839 (MRSA)	5	1.25	10-20
**9.**	GMCH 6152(MRSA)	20	5	10-20
**10.**	GMCH 3939 (MRSA)	10	2.5	10-20

Table S3: Showing Information regarding cultures

Strain	MIC (µg/mL)			
	Oxacillin	Vancomycin	Ciprofloxacin	Erythromycin
**ATCC 29213**	0.25	1	0.5	0.25
**ATCC 25923**	≤1	≤1	≤1	0.125
**ATCC 43300**	64	1	≤1	0.25
**ATCC 33591**	>128	1	0.5	>128
**ATCC-BAA-39**	64	1	8	>256
**GMCH 839 ***	2	1	8	>128
**GMCH 6152 ***	16	2	32	≤1
**GMCH 3939***	4	1	>16	>128
**MRSA-1***	4	1	8	>500
**MRSA-4***	4	1	32	>128

*Clinical isolates acquired from GMCH-32 Chandigarh, 160036, INDIA.

Table S4: Comparison of various ciprofloxacin-based drug delivery platforms along with SL- based niosome delivery system: physicochemical characteristics and target pathogens

Formulation	composition	Method of preparation	Particle Size	PDI	Zeta potential	Entrapment efficiency	Target pathogen	Key advantage	Reference
**PLGA (Poly (DL-lactide-co-glycolide) microsphere**	Ciprofloxacin, PLGA	Double emulsion solvent evaporation (w/o/w)	10 to 50 µm	-	-	60–70%	*Staphylococcus aureus*, *Escherichia coli*	Active against *Staphylococcus aureus*, *Escherichia coli*, etc.; comparable or slightly better than free ciprofloxacin in zone of inhibition tests	[1]
**Sophorolipid based niosome**	Sophorolipid, Cholesterol,Steryl amine,	Thin film layer	269.9 ± 31.5 nm	0.27 ± 0.04	51.13 ± 6.99	71	MRSA	Biofilm Reduction in Resistant strain	Present study
**Solid lipid nanoparticles**	Softisan ,Dynasan Imwitor stearic acid (STE), CIP	ultrasonic melt-emulsification method	165 to 320 nm	0.18 – 0.33	-25 to -30mV	70-85%	*Escherichia coli*	Enhanced antibacterial activity compared to ciprofloxacin	[2]
**Chitosan**	Chitosan, ciprofloxacin Cholesterol span 60	Ionic gelation / Reverse phase evaporation / Thin film hydration	180.34 ± 5.13 nm	0.221	25 ± 2 mV	78.32 ± 4.49%	*Pseudomonas aeruginosa* *S. aureus*	Ciprofloxacin-loaded vesicles showed inhibition zones of [e.g., 20 mm], comparable or superior to free drug	[3]
**Liposomes**	Ciprofloxacin HCl, phosphatidylcholine-based (e.g. HSPC / likely egg PC), Farnesol, Cholesterol	Thin-film hydration followed by extrusion	160–180 nm		30 mV	65–70%	*Pseudomonas aeruginosa* (biofilm-forming clinical isolates) |	Farnesol synergistically enhances liposomal ciprofloxacin's biofilm penetration and antimicrobial efficacy	[4]
**Chitosan**	CS, TPP, CHCL	ionic gelation technique	198–304	0.02-0.3	+27 to +42 mV	23–45%	*Pseudomonas aeruginosa* and *Staphylococcus aureus*	MIC against *Pseudomonas aeruginosa* and *Staphylococcus aureus* reduced by 4-fold compared with pure ciprofloxacin HCl	[5]

References:

1. Psimadas, D.; Georgoulias, P.; Valotassiou, V.; Loudos, G. Molecular Nanomedicine Towards Cancer : *J. Pharm. Sci.* **2012**, *101*, 2271–2280, doi:10.1002/jps.

2. Shazly, G.A. Ciprofloxacin Controlled-Solid Lipid Nanoparticles: Characterization, in Vitro Release, and Antibacterial Activity Assessment. *Biomed Res. Int.* **2017**, *2017*, doi:10.1155/2017/2120734.

3. Ameeduzzafar; Alruwaili, N.K.; Imam, S.S.; Alotaibi, N.H.; Alhakamy, N.A.; Alharbi, K.S.; Alshehri, S.; Afzal, M.; Alenezi, S.K.; Bukhari, S.N.A. Formulation of Chitosan Polymeric Vesicles of Ciprofloxacin for Ocular Delivery: Box-Behnken Optimization, In Vitro Characterization, HET-CAM Irritation, and Antimicrobial Assessment. *AAPS PharmSciTech* **2020**, *21*, doi:10.1208/s12249-020-01699-9.

4. Bandara, H.M.H.N.; Herpin, M.J.; Kolacny, D.; Harb, A.; Romanovicz, D.; Smyth, H.D.C. Incorporation of Farnesol Significantly Increases the Efficacy of Liposomal Ciprofloxacin against Pseudomonas Aeruginosa Biofilms in Vitro. *Mol. Pharm.* **2016**, *13*, 2760–2770, doi:10.1021/acs.molpharmaceut.6b00360.

5. Soliman, N.M.; Shakeel, F.; Haq, N.; Alanazi, F.K.; Alshehri, S.; Bayomi, M.; Alenazi, A.S.M.; Alsarra, I.A. Development and Optimization of Ciprofloxacin HCl-Loaded Chitosan Nanoparticles Using Box–Behnken Experimental Design. *Molecules* **2022**, *27*, doi:10.3390/molecules27144468.
